# Antibacterial coating of implants in orthopaedics and trauma: a classification proposal in an evolving panorama

**DOI:** 10.1186/s13018-015-0294-5

**Published:** 2015-10-01

**Authors:** Carlo Luca Romanò, Sara Scarponi, Enrico Gallazzi, Delia Romanò, Lorenzo Drago

**Affiliations:** Department of Reconstructive Surgery of Osteo-articular Infections C.R.I.O. Unit, IRCCS Galeazzi Orthopaedic Institute, Via R. Galeazzi 4, 20161 Milan, Italy; Laboratory of Clinical Chemistry and Microbiology, I.R.C.C.S. Galeazzi Orthopaedic Institute, Milan, Italy

**Keywords:** Orthopaedics, Trauma, Implant, Joint, Prosthesis, Biofilm, Infection, Coating, Antibacterial, Classification

## Abstract

Implanted biomaterials play a key role in current success of orthopedic and trauma surgery. However, implant-related infections remain among the leading reasons for failure with high economical and social associated costs. According to the current knowledge, probably the most critical pathogenic event in the development of implant-related infection is biofilm formation, which starts immediately after bacterial adhesion on an implant and effectively protects the microorganisms from the immune system and systemic antibiotics. A rationale, modern prevention of biomaterial-associated infections should then specifically focus on inhibition of both bacterial adhesion and biofilm formation. Nonetheless, currently available prophylactic measures, although partially effective in reducing surgical site infections, are not based on the pathogenesis of biofilm-related infections and unacceptable high rates of septic complications, especially in high-risk patients and procedures, are still reported.

In the last decade, several studies have investigated the ability of implant surface modifications to minimize bacterial adhesion, inhibit biofilm formation, and provide effective bacterial killing to protect implanted biomaterials, even if there still is a great discrepancy between proposed and clinically implemented strategies and a lack of a common language to evaluate them.

To move a step forward towards a more systematic approach in this promising but complicated field, here we provide a detailed overview and an original classification of the various technologies under study or already in the market. We may distinguish the following: 1. Passive surface finishing/modification (PSM): passive coatings that do not release bactericidal agents to the surrounding tissues, but are aimed at preventing or reducing bacterial adhesion through surface chemistry and/or structure modifications; 2. Active surface finishing/modification (ASM): active coatings that feature pharmacologically active pre-incorporated bactericidal agents; and 3. Local carriers or coatings (LCC): local antibacterial carriers or coatings, biodegradable or not, applied at the time of the surgical procedure, immediately prior or at the same time of the implant and around it. Classifying different technologies may be useful in order to better compare different solutions, to improve the design of validation tests and, hopefully, to improve and speed up the regulatory process in this rapidly evolving field.

## Introduction

Even if current peri-operative infection prevention methods, like antibiotic prophylaxis, have significantly reduced the incidence of surgical site infections (SSI), up to 2.5 % of primary hip and knee arthroplasties and to 10 % of revision arthroplasties can still be complicated by periprosthetic joint infection (PJI) [1]. Moreover, according to recent analysis, these figures could even be underestimated and are on the rise [2], while multi-resistant pathogens are often retrieved [3]. The occurrence of PJI is a devastating complication, often requiring implant removal, with high morbidity and possible mortality raise [4] and elevated economical and social costs [5].

Implant-related infections are the results of a complex interaction of various factors, including bacterial load, microorganism’s and host’s type, surgical procedure and technique, and type of implant and of antibacterial prophylaxis.

In fact, even elective surgery may not be performed in a completely sterile environment, and operating rooms have been shown to become contaminated within the first few hours of service [6, 7]. While in the majority of cases the rather low bacterial load eventually present at surgery may be generally overcome by the host’s immunological defense and the systemic antibiotic prophylaxis [8], in some patients a SSI may eventually develop, especially in high-risk patients, in which relevant co-morbidities may increase the relative risk for infection up to 20 times compared to normal population [9–11]. Similarly, the most complex surgical procedures and techniques have been shown to be more prone to septic complications [12]. In this context, implant features, including size, shape, material, and intended use, also play an important role [13], while, for example, local antibacterial prophylaxis, like the use of antibiotic-loaded bone cement or bone grafts has been shown to reduce the incidence of implant-related infections [14, 15]. In line with these considerations, a strong recommendation was delivered, in a recent international consensus meeting on PJI, concerning the need for developing effective antibacterial surfaces that prevent bacterial adhesion and colonization of implants and proliferation into the surrounding tissues [16].

In the present review, we provide a detailed description and an original classification of the technologies already available or under investigation, aimed at minimizing implant-related infections in orthopedics and trauma surgery.

## Rationale of antibacterial coating of implants

Prophylactic systemic antibiotics are administered routinely to patients who receive an orthopedic device to prevent peri-operative infection [8]. However, systemic administration of antibiotics has many potential disadvantages including the need for a correct timing of administration, the relatively low drug concentration at the target site, and the limited ability to kill bacteria eventually present on the implant surface or embedded in biofilms.

When Anthony Gristina first proposed the concept of a “race for the surface”, more than three decades ago, he described a simplified model of implant-related infection, whereby host and bacterial cells compete in determining the ultimate fate of the implant [17]. According to this model, when the host cells colonize the implant surface first, the probability of attachment of bacterial cells is very low and vice versa. Further studies have now made it clear that the process is much more complex and only partially understood.

Bacteria have a highly successful and diversified strategy to adhere and survive on virtually all natural and synthetic surfaces [18, 19]. Surface characteristics of a biomaterial such as roughness, hydrophobicity, and electrostatic charge play only conditional roles [20], while a number of potential receptors for bacterial adhesive ligands are offered by the protein film that covers an implant immediately after its placement into the host body [21–23]. Complement, albumin, and several other host proteins and lipids are the main components of this conditional protein film [24–26]. The process of bacterial adhesion can be divided into a reversible phase, based on nonspecific interactions between implant surface and bacterial adhesions, and an irreversible phase, mediated by molecular and cellular interactions and closely associated with expression of biofilm-specific gene clusters in reversibly attached bacteria. All the process from bacterial adhesion to the production of a mature biofilm is extremely efficient and is normally completed within 12 to 18 h [27–30].

On the host side, the detailed process of implant osteo and tissue integration is also incompletely unveiled [31, 32]. According to Gristina’s model, host cells, once attached to implant surfaces, should lead to periprosthetic bone regeneration and remodeling, protecting the biomaterial against bacterial colonization [33]. However, bacteria may survive to osteointegration or to fibrous tissue encapsulation of an implant, while peri-implant fibrous tissue may even prevent direct contact between host immunity cells and bacterial molecules, while the presence of the implant has been shown to impair innate local host response [34–36].

As a result, there is a strong need for intrinsic implant surface antibacterial functionality to overcome the implant-induced defects in the local immune response and to prevent the striking ability of bacteria to quickly adhere on a substrate and immediately produce a protective biofilm barrier, providing a competitive advantage to the host’s cells over the contaminating microorganisms. This is of utmost importance especially in patients with underlying compromised immunity [37] and in those undergoing revision surgery [38] in which the relative risk of infection is multiplied.

At the same time, any coating technology should prove to be safe in the short and long term, should not interfere with osteointegration or induce bacterial resistance in the long run, and should be easy to implement in the clinical practice and at an affordable cost. Also, since bacterial colonization, from microbial adhesion to an established mature biofilm layer only takes few hours [39], any antibacterial protection should act at the exact time of surgery and for at least some hours or days thereafter.

## Classification of antibacterial coating technologies

Local antibacterial implant protection can be achieved in different ways. Currently, there is not a single, universally accepted classification of coating technologies, standardized validation methods are lacking and regulatory aspects appear somewhat inadequate in view of the clinical needs and expectations. Table 1 summarizes the basic requirements that an “ideal” coating technology should fulfill to meet the needs of a widespread clinical use.

**Table 1:** A list of requirements to be fulfilled by the “ideal” antibacterial implant coating strategy

Requirements	Fulfillments			
Safety	No systemic toxicity	No local toxicity	No detrimental effects on bone healing	No unwanted long-term side effects
In vitro activity	No cytotoxicity or genotoxicity	Proven bactericidal and antibiofilm activity on different surfaces	Large spectrum	No induction of resistance
Efficacy	Proven in vivo	Case series	Multicenter trials	Randomized trials
Ease-of-use	Easy handling	Versatility	Resistance to press-fit insertion	Storage
Market	Acceptable cost	Large availability	Easy to manufacture	Overcomes regulatory issues

According to their strategy of action, antibacterial coatings can be classified in at least three groups (Table 2):

**Table 2:** Classification of antibacterial implant protection strategies

Strategy	Features	Examples	Development stage	Limits
Passive surface finishing/modifications (PSM)	Prevention of bacterial adhesion	Hydrophilic surface	Preclinical	Limited antibacterial and antibiofilm activity
		Super-hydrophobic surface		
		Anti-adhesive polymers		Possible interference with osteointegration
		Nano-patterned surface		
		Albumin		Unknown long-term effects
		Hydrogels		Regulatory issues
		Biosurfactants		
Active surface finishing/modifications (ASM)	Inorganic	Silver ions and nanoparticles	Market	Incomplete implant coating
				Questionable long-term toxicity
				Limited versatility and applicability
				Limited large-scale applications
				Possible bacterial resistance induction
				Costs
		Other metals (copper, zinc, titanium dioxide etc.)	Preclinical	Questionable long-term toxicity
				Regulatory issues
		Non-metals: Iodine	Clinical	Incomplete implant coating
				Questionable long-term toxicity
				Challenging large-scale application
				Regulatory issues
		Other non-metal ions (selenium, grapheme, etc.)	Preclinical	Poorly studied compounds
				Coating resistance to press-fit insertion
				Questionable long-term toxicity
				Challenging large-scale application
				Regulatory issues
	Organic	Coated/linked antibiotics	Market	Unique application to nail coating
				Long-term effects on osteointegration
				Single antibiotic (gentamicin)
		Covalently linked antibiotics	Preclinical	Incomplete implant coating.
				Questionable long-term toxicity
				Challenging large-scale application
				Regulatory issues
		Antimicrobial peptides		No data on in vivo or clinical effects
		Cytokines		Coating resistance to press-fit insertion
		Enzymes and biofilm disrupting agents		Questionable long-term toxicity
				Challenging large-scale application
		Chitosan derivatives		
	Synthetic	Non-antibiotic antimicrobial compounds		
				Regulatory issues
		“Smart” coatings		
	Combined	Multilayer coating		
Peri-operative antibacterial local carriers or coatings (LCC)	Not-biodegradable	Antibiotic-loaded polymethylmetacrylate	Market	Resistance and small-colony variants induction
				No antibiofilm effect
				Incomplete implant coating
				May not be used for cementless implants
	Biodegradable	Antibiotic-loaded bone grafts and substitutes	Market	Limited availability
				Not proven efficacy as implant coating
				Cost
		Fast-resorbable hydrogel	Market	Early clinical use and results

1. Passive surface finishing/modification (PSM). Here all surface chemistry and/or structure modifications aimed at preventing or reducing bacterial adhesion, without releasing bactericidal agents to the surrounding tissues, are included.
2. Active surface finishing/modification (ASM). Active coatings, included in this class, feature pharmacologically active pre-incorporated antibacterial agents, like antibiotics, antiseptics, metal ions, or other organic and inorganic compounds.
3. Peri-operative antibacterial local carriers or coatings (LCC). According to this strategy, implant protection is achieved through a biodegradable or not-biodegradable antibacterial carrier or coating, applied during surgery on the implant or around it; the coating may simply act by delivering high local concentrations of one or more pre-loaded antibacterials, or it may have a direct or synergistic antibacterial activity. Antibiotic-loaded polymethylmethacrylate is probably the very first example of this coating strategy, used since many years to protect joint implants.

Although necessarily schematic, this classification may be helpful to compare different technologies both in the clinical practice and concerning regulatory aspect, that should probably differ for different classes.

### Passive surface finishing/modification

Chemical and/or physical modifications of the surface layer of an existing biomaterial may result in a substantial change of its susceptibility to bacterial colonization.

Surface characteristics of implants, like surface roughness and chemistry, hydrophilicity, surface energy or potential and conductivity play in fact crucial roles in bacterial adhesion and subsequent biofilm formation. Surface physio-chemical modification of an implant is also relatively simple and economic to achieve and to industrialize.

Ultraviolet light irradiation, for example, is able to increase “spontaneous” wettability on titanium dioxide, which in turn can inhibit bacterial adhesion without compromising osteogenesis on titanium alloy implants [40, 41]. A bacterial anti-adhesive surface can also be achieved by modifying the crystalline structure of the surface oxide layer [42].

Some polymer coatings, like the hydrophilic polymethacrylic acid, polyethylene oxide, or protein-resistant polyethylene glycol can be applied to the surface of titanium implants and result in significant inhibition of bacterial adhesion [43–45]. Even if some of these coatings may impair local osteoblast function, the use of additional bioactive molecules as sericin and RGD motif could restore and even improve the impaired cell function [46]. Hydrophobic and super-hydrophobic surface treatment technologies have also shown a great repellent antibacterial effect in preclinical studies [47, 48].

Other researchers have focused on controlling the biological response to biomaterials via alterations in surface structure and design [49, 50]. In this regard, changing the implant surface at a nanometric scale, at which bacterial adhesion does not simply follow the roughness of the surface but also is dependent on other variables like the quantity of adsorbed proteins, can in fact suppress bacteria adhesion [51].

Treating more specifically protein surfaces and/or protein–bacteria interactions may also be a successful strategy of inhibiting bacterial adhesion to a specific biomaterial [52, 53]. Friedman et al*.*, using a rabbit model, demonstrated reduced bacterial adherence on pure titanium samples and decreased infection rates of implants coated with cross-linked albumin [54].

More recently, novel strategies include production of self-assembled mono- or multilayers, surface grafting or hydrogels, or the use of biosurfactants and microbial amphyphilic compounds with excellent anti-adhesive properties [55, 56].

In summary, a number of anti-adhesive surface modifications have been proposed for different purposes, but only a few will probably be suitable for clinical use. In particular, a strong anti-adhesive layer cannot be used for coating of fixation surfaces of joint arthroplasty since it could also interfere with implant osteointegration, leading to early mechanical failure [48, 49]. Another challenge of designing anti-adhesive technologies relates to the current inability to find a universal treatment that can be applied to all surfaces and biomaterials, all bacterial species and under all (ingrowth and noningrowth) implants. Moreover, passive coating methods should be preferred as long as their antibacterial ability is strong enough to prevent biofilm formation, but the ability of passive coatings to resist bacterial adhesion is generally limited and varies greatly depending on the bacterial species and loads [57]. In vivo efficacy and long-term effects of these new technologies both on host’s cells and on bacterial resistance are also poorly understood and need to be further investigated before clinical applications and market introduction.

### Active surface finishing/modification

Surface modifications may include pharmacologically active pre-incorporated antibacterial agents or compounds, like antibiotics, antiseptics, metal ions, or organic molecules. Such pharmacologically activated coatings may change the implant from a passive, pharmacologically inert medical device, to something more and more similar to a drug agent, with difficult to predict long-term effects and challenging regulatory issues.

Historically, two main strategies have been proposed for effective antibacterial surface treatment either “contact killing” or drug eluting, while in terms of durability, we can distinguish between degradable and non-degradable coatings. Killing of bacteria can be achieved by interfering with cell respiration or division, cell wall formation or bacterial signaling network as well as inhibition of the transition of planktonic phenotype of bacteria into a sessile type [58].

Antibacterial surface technologies can employ metals (silver, zinc, copper, etc.), non-metal elements (e.g., iodine, selenium), organic substances (antibiotics, anti-infective peptides, chitosan, other substances), and their combinations.

Antibacterial activity of the majority of metal coatings is closely linked to the ionic or nano form rather than to the bulk material [59]. Silver is the most prevalent metal used in biomedical applications. Dissolved silver cations are biochemically active agents that interfere with bacterial cell membrane permeability and cellular metabolism. Silver also contributes to formation of reactive oxygen species and other mechanisms that potentially influence prokaryotic cells [60]. There has been concern, however, about the toxicity of silver ions [61]. Research efforts have focused on the development of silver coating technologies that reduce or even eliminate toxicity while maintaining antibacterial effects [62, 63]. Despite demonstrated clinical efficacy and safety in recent comparative studies [64, 65], routine use of silver-coated implants remains rather limited. The main obstacle to a broader usage of such technology is cytotoxicity on bone cells, that prevented until now coating of the intra-medullary part of the prosthesis. In addition, cost issues and the inability to apply the technology to a variety of prosthetic implants and devices further reduces its application outside oncological or highly selected cases.

Copper and zinc also have potent antibacterial effects on a wide spectrum of bacterial species [66, 67]; however, potential toxic side effects of these metals remain a strong concern [68]. Proposed solutions include copper- and zinc-based nanomaterials or, alternatively, controlled release [69]. The risk of bacterial resistance to metallic coatings remains a potential limitation for their widespread use [70]. Concern also exists about the mechanical properties of implant nanocoatings since damage may occur during surgical implantation especially in cementless implants inserted via press-fit methods [71].

Another interesting technology is related to modification of commonly used alloys, like titanium. The anti-infective potential of titanium dioxide layers has been widely investigated and proven effective in vitro both alone [72] or in combination with other substances [73].

Non-metal elements like hydrogen, chlorine, iodine, or oxygen are commonly used in biomedicine for their anti-infective properties. Selenium bound covalently onto the surface of titanium or titanium alloy implant discs has been shown to prevent *Staphylococcus aureus* and *Staphylococcus epidermidis* attachment without affecting osteoblast viability [74]. Selenium catalyzes the formation of superoxide radicals and subsequently inhibits bacterial adhesion and viability. In addition, selenium nanoparticles can inhibit bacterial growth and biofilm formation [75].

Ongoing research is also directed to determine the clinical applicability of carbon substances like graphene or carbon nanotubes, that can be synthesized in multifunctional layers [76]; however, the most interesting technology today under study, related to non-metal elements, is probably iodine coating of titanium alloys, that has recently demonstrated clinical efficacy in a continuous series of 222 patients with excellent results [77].

Several organic compounds with antibacterial properties have the potential to be linked to the surface of implants conferring them anti-infective properties. A large number of studies have investigated the efficacy of surfaces coated with covalently linked antibiotics [78–82]. Clinical effectiveness of such implants is most likely limited to infections caused by bacteria that are sensitive to the specific antibiotic that has been coupled. In addition, strong forces such as covalent binding are insufficiently sensitive to react to weak external stimuli [83]. In fact, despite the theoretical advantages for non-eluting systems, this concept is limited by the fragility of the coatings and killing activity potential of bacteria which might not be directly adjacent to the implant. To overcome these issues, combinations of antibiotics with other compounds have been proposed either alone or in association with a particular mechanism of controlled release [84]. Antibiotics such as gentamicin, vancomycin, and others have been loaded into porous hydroxyapatite (HA) coatings on titanium implants. The antibiotic-HA coatings exhibit significant improvement in preventing infection compared with standard HA coatings in vivo, but there are still many unresolved issues regarding the methodology of antibiotic incorporation into the HA coating and the optimal release kinetics and possible detachment of the coating at the time of press-fit insertion.

Biodegradable polymers and sol–gel coatings are also utilized to form controlled release antibiotic-laden coatings on titanium implants [85, 86]. Clinical applications of antibiotic-loaded D-poly-lactate acid/gentamycin intra-medullary coated nail have been recently reported with early positive results [87].

Some antiseptic agent such as chlorhexidine, chloroxylenol, or poly-hexamethylenebiguanide have demonstrated efficacy and might be an alternative to avoid the risk of drug resistance. Chlorhexidine can be adsorbed to the TiO2 layer on titanium surfaces and is released gradually over several days [88]. Its release pattern is similar to that of antibiotic-laden coatings with an initial rapid release rate followed by slower but sustained release [89].

Another promising approach involves coating implants with antimicrobial peptides, cytokines, or other molecules critical for host response to bacteria invasion. This heterogeneous group of substances has proven experimentally their efficacy against a wide range of pathogens [90]. Antimicrobial peptides, like antibiotics, function via damage of the cell wall and inhibition of key bacterial protein synthesis. In addition, they exert influence upon inflammation, tissue healing, and apoptotic events [91]; resistance to antimicrobial peptides has been reported less frequently than to antibiotics [92]. Initial experiments demonstrated that a thin layer of antimicrobial peptides affixed onto the surfaces of metal alloys exhibit excellent antibacterial effects against typical pathogens related to PJI [93].

Chitosan (CS) is a polycationic polymer derived from chitin that exhibits antibacterial and antifungal activity. The exact mechanism of action remains poorly understood. There is some evidence that CS derivatives can be firmly anchored to titanium alloys and that they have a protective effect against some bacterial species either alone or in combination with other antimicrobial substances like antibiotics or antimicrobial peptides [94, 95]. CS derivatives secured to external fixator pins have been studied as a method of preventing pin tract infections [96]. However, we are not aware of a study to date reporting data from clinical setting.

Long-term impact of permanently coated implants with antibiotics and other organic compounds, never used before either for local or general administration, does raise concerns regarding possible induction of bacterial resistance, local, and general toxicity and possible detrimental effects on implant osteointegration, ultimately preventing clinical applications until now.

Still more complex approaches involve the development of multifunctional surface layers, like functional polymer brush coating, that combine anti-adhesive and antimicrobial substances and other compounds able to enhance tissue integration [97], while “smart coatings”, sensitive and responsive to a variety of stimuli, including the presence of bacteria [98], are another fascinating but futuristic research pathway that poses a number of open questions, like feasible coating manufacturing process, non-adverse reactions in vivo, mechanical resistance, or preservation of intended functionalities throughout the life of the device.

### Peri-operative antibacterial carriers or coatings

Instead of pre-manufactured surface modifications, either with or without pharmacologically active agents, a different approach to implant protection may be to provide a traditional implant with an antibacterial carrier or coating at the time of surgery. The separation of the protective solution from the implant until surgery may reduce the regulatory requirements and increase the applicability of a universal antibacterial coating to many different already existing implants and biomaterials.

Local administration of antibiotics historically attracted much attention in orthopaedics. Buchholz et at. first popularized the incorporation of antibiotics into polymethylmethacrylate (PMMA) bone cement for local antibiotic prophylaxis in cemented total joint arthroplasty [99]. Clinical studies have shown that antibiotic-loaded bone cement can decrease deep infection rates of cemented total hip arthroplasties and revision rates due to supposed “aseptic” loosening when combined with systemic antibiotic administration [100] and this solution has been found both effective and economically sound, especially in high-risk patients [101, 102]. However, PMMA was not designed as a local delivery carrier of antibiotics and may have some limits. Antibiotic-loaded PMMA may not overcome biofilm formation and may be associated with the development of antibiotic-resistant “small-colony variants” [103, 104], while the increasing use of cementless implants worldwide, especially at the hip site, make this a possible option only for a restricted number of patients.

Other porous materials for local antibiotic delivery like collagen sponges [105], cancellous bone [106], and calcium phosphate cements [107, 108] were not specifically designed to protect implanted biomaterials, and their use for routine infection prevention in joint prosthesis is limited by their insufficient in vitro, in vivo, and clinical evidence of efficacy in this specific application, their inability to be applied as a coating to all implants’ surfaces, and their relatively high costs and possible interference with primary implant fixation and long-term osteointegration.

**Fig. 1:**
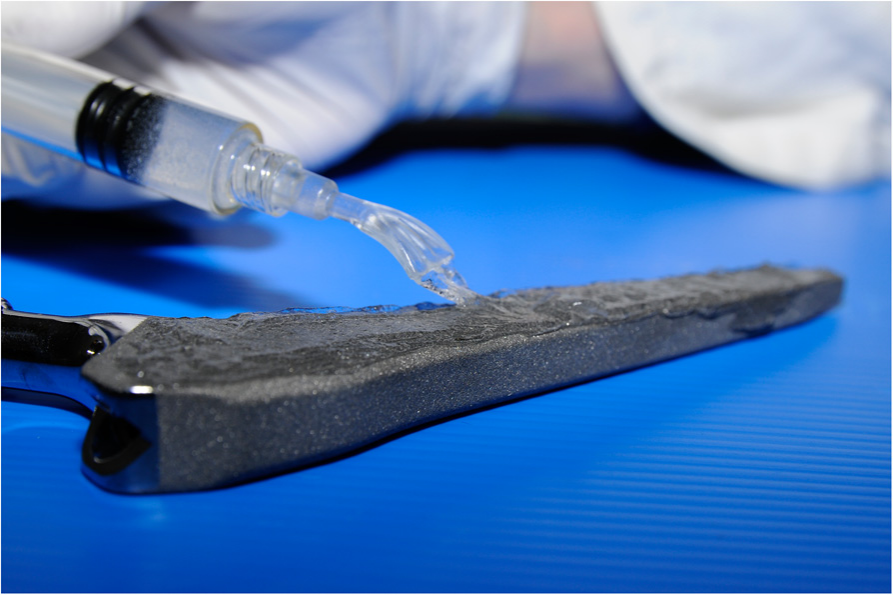
“Defensive Antibacterial Coating”, DAC® (Novagenit Srl, Mezzolombardo, Italy): a fast-resorbable hydrogel coating, composed of covalently linked hyaluronan and poly-D,L-lactide, is spread onto a cementless hip prosthesis. The hydrogel is loaded intra-operatively with one or more antibiotics that are released within 48 to 72 h, providing antibacterial and antibiofilm protection to the implant

**Fig. 2:**
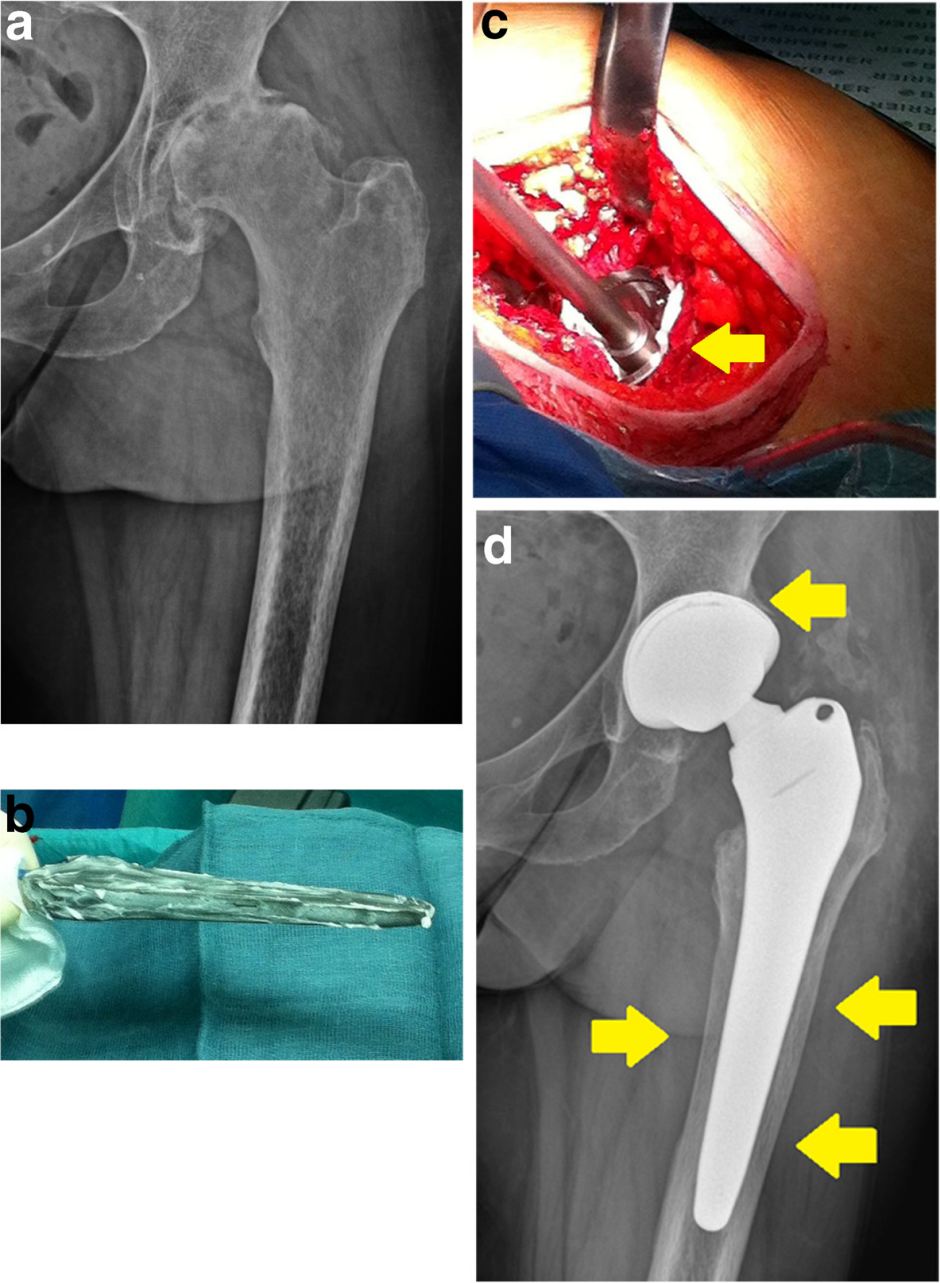
**a** Radiographic antero-posterior view of sequelae of a septic hip arthritis (*S. aureus*) in a 52-year-old female patient. **b** Teicoplanin 5 % loaded DAC® hydrogel is applied on the sanded titanium surface of a standard cementless hip prosthesis, both on the stem and **c** in the acetabulum component that may be press-fit inserted according to a normal procedure (*arrow* indicates some hydrogel squeezed out during cup insertion). **d** Control after 12 months shows optimal bone osteointegration (*arrows*). The patient is pain- and infection-free

Biocompatible hydrogels do represent a possible alternative solution, as they have demonstrated to be able to deliver local pharmacological agents and may be designed to meet the desired elution pattern [109]. Recently, a fast-resorbable hydrogel coating, that can be loaded intra-operatively with various antibacterials, has been introduced in the European market [110]. Based on the facts that bacterial colonization occurs within the very first hours after implant and that short-term systemic prophylaxis is equally effective as the long-term one to prevent PJIs [111], this novel coating technology introduced the “short-term local protection” of the implant. A short-term local delivery system may in fact meet the requirements needed to win the “run to the surface”, while limiting possible long-term unwanted side effects [112]. This novel fast-resorbable hydrogel coating, “Defensive Antibacterial Coating”, DAC® (Novagenit Srl, Mezzolombardo, Italy), composed of covalently linked hyaluronan and poly-D,L-lactide, (Fig. 1), is designed to undergo complete hydrolytic degradation in vivo within 48 to 72 h, being able to completely release a variety of different antibacterials, including glycopeptides, amynoglycosides, and fluoquinolones (Table 3), at concentrations ranging from 2 % to 10 %. The hydrogel showed a synergistic antibacterial activity with various antibiotics and antibiofilm agents in vitro [113], while in vivo it has been proven effective a rabbit model of highly contaminated implant both with [114] and without systemic prophylaxis [115]. An ongoing European multicenter clinical trial (Fig. 2) is currently investigating the safety and the efficacy of the device [116].

**Table 3:** Tested antibacterials to be loaded with DAC hydrogel coating at concentrations ranging from 2 to 10 % ([113] and Novagenit Srl data on file)

Antibacterial family	Tested antibiotics
Aminoglycosides	Gentamicin
	Tobramycin
	Amikacin
Carbapenems	Meropenem
Glicopeptides	Vancomycin
	Teicoplanin
Quinolones	Ciprofloxacin
Cyclic lipopeptides	Daptomycin
Rifamycins	Rifampicin
Glycylcyclines (Tetracyclines)	Tigecyclin
Oxazolidinones	Linezolid
Antifungals	Amphotericin B
	Fluconazole
	Ketoconazole

## Conclusions

A tremendous amount of research has been done in the last two decades in order to better protect implanted biomaterials from bacterial colonization, but we are still probably at the beginning of the journey. In fact, in spite of the highly successful advances in preclinical studies, a remarkable discrepancy still exists between the proposed strategies and their clinical applications [117, 118]. While most of the coatings under study will not probably be suitable for orthopedic implants, due to cytotoxicity, immunoreactivity, or genotoxicity problems, clinical application of those successfully tested in vitro and in vivo can still be limited by biotechnological, regulatory, economic, and medico-legal issues.

Improving collaborative efforts amongst governments, regulatory agencies, industry leaders, and health care payers will probably allow more and more our patients to benefit from these very promising technologies.

A comprehensive classification and a common language are needed to standardize appropriate tests and realistic regulatory requirements, in order to favor the transition from preclinical studies to effective patient’s protection.

## Consent

Written informed consent was obtained from the patient for the publication of this report and any accompanying images.
